# Investigation of the urinary sodium-to-potassium ratio target level based on the recommended dietary intake goals for the Japanese population: The INTERMAP Japan

**DOI:** 10.1038/s41440-022-01007-x

**Published:** 2022-11-08

**Authors:** Ebtehal Salman, Aya Kadota, Yukiko Okami, Keiko Kondo, Katsushi Yoshita, Nagako Okuda, Hideaki Nakagawa, Shigeyuki Saitoh, Kiyomi Sakata, Akira Okayama, Queenie Chan, Paul Elliott, Jeremiah Stamler, Hirotsugu Ueshima, Katsuyuki Miura, for the INTERMAP Research Group

**Affiliations:** 1 NCD Epidemiology Research Center, Shiga University of Medical Science, Shiga, Japan; 2 OMRON Healthcare Co., Ltd, Kyoto, Japan; 3 Department of Food Science and Nutrition, Graduate School of Human Life Science and Nutrition, Osaka City University, Osaka, Japan; 4 Department of Health Science, Kyoto Prefectural University, Kyoto, Japan; 5 Medical Research Institute, Kanazawa Medical University, Kanazawa, Japan; 6 School of Health Sciences, Sapporo Medical University, Sapporo, Japan; 7 Department of Hygiene and Public Health, Iwate Medical University, Yahaba, Japan; 8 Department of Non-Communicable Diseases, Research Institute of Strategy for Prevention, Tokyo, Japan; 9 Department of Epidemiology and Biostatistics, School of Public Health, Imperial College London, London, UK; 10 Department of Preventive Medicine, Feinberg School of Medicine, Northwestern University, Chicago, IL USA

**Keywords:** Cutoff level, Dietary guidelines, Japanese, Urinary sodium-to-potassium

## Abstract

Growing epidemiological evidence has shown an association of the urinary sodium (Na) to potassium (K) ratio (Na/K ratio) with blood pressure and cardiovascular diseases. However, no clear cutoff level has been defined. We investigated the cutoff level of the urinary Na/K ratio under different dietary guidelines for Japanese individuals, especially that endorsed by the 2020 revised Japanese Dietary Reference Intakes (DRIs). A population of 1145 Japanese men and women aged 40 to 59 years from the INTERMAP study was examined. Using high-quality standardized data, the averages of two 24 h urinary collections and four 24 h dietary recalls were used to calculate the 24 h urinary and dietary Na/K ratios, respectively. Associations between the urinary and dietary Na/K ratios were tested by sex- and age-adjusted partial correlation. The optimal urinary Na/K ratio cutoff level was determined by receiver operating characteristic (ROC) curves and sex-specific cross tables for recommended dietary K and salt. Overall, the average molar ratio of 24 h urinary Na/K was 4.3. We found moderate correlations (*P* < 0.001) of the 24 h urinary Na/K ratio with 24 h urinary Na and K excretion (*r* = 0.52, *r* = −0.49, respectively) and the dietary Na/K ratio (*r* = 0.53). ROC curves showed that a 24 h urinary Na/K ratio of approximately 2 predicted Na and K intake that meets the dietary goals of the Japanese DRIs. The range of urinary Na/K ratios meeting the dietary goals of the Japanese DRIs for both Na and K was 1.6‒2.2 for men and 1.7‒1.9 for women. Accomplishing a urinary Na/K ratio of 2 would be desirable to achieve the DRIs dietary goals for both Na and K simultaneously in middle-aged Japanese men and women accustomed to Japanese dietary habits. This observational study is registered at www.clinicaltrials.gov as NCT00005271.

## Abstract (web summary)

Our proposition is accomplishing cutoff value of approximately 2 for urinary Na/K ratio would be desirable to meet the DRIs dietary goals of both Na and K simultaneously in middle-aged Japanese men and women accustomed to Japanese dietary habits.

## Graphical abstract

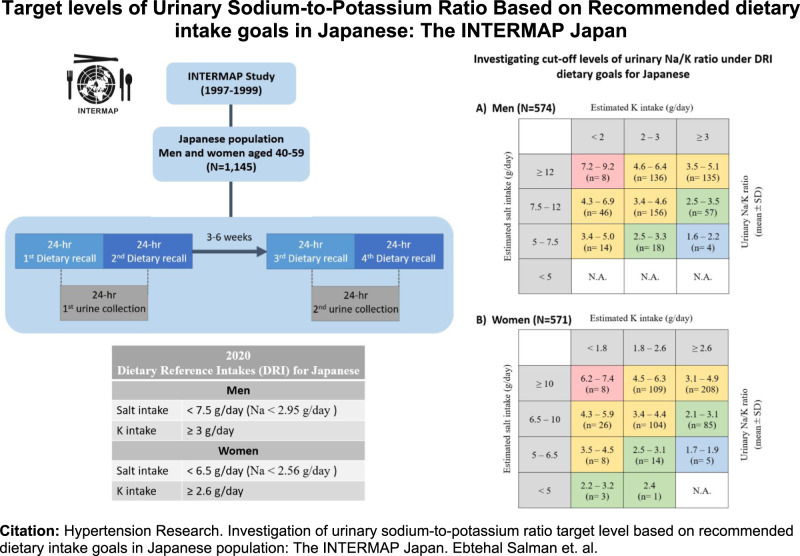

## Introduction

Regardless of whether 24 h measurement or casual spot urine is used, repeated urinary collection is considered an objectively reliable measure to estimate an individual’s habitual consumption level of sodium (Na) and potassium (K) from the excreted Na and K in the urine, respectively [1–5]. Cumulative evidence has shown that the sodium-to-potassium (Na/K) ratio is a more important measure in relation to blood pressure (BP) and cardiovascular diseases (CVDs) than Na and K alone [2, 6–9]. Several studies have shown a linear positive relationship between the urinary Na/K ratio and hypertension and CVDs [6, 8–14]. Therefore, monitoring the urinary Na/K ratio is a useful and valid estimate of the dietary intake of Na and K as a method for disease prevention.

Thus far, established dietary guidelines have identified intake goals for Na and K among different populations. In April 2020, the Japanese Ministry of Health, Labor and Welfare revised the Dietary Reference Intakes (DRIs) for Japanese individuals and set the dietary goal for salt intake to be less than 7.5 g/day for men (Na < 2.95 g/day [128.3 mmol/day]) and less than 6.5 g/day for women (Na < 2.56 g/day [111.3 mmol/day]) [15]. The daily K intake goal according to the DRIs did not change from the previous guideline, which was set to at least 3 g/day for men (77 mmol/day) and at least 2.6 g/day for women (67 mmol/day) [15]. Other dietary guidelines differ regarding the dietary intake goal of Na and K. The 2012 World Health Organization (WHO) guidelines recommend a Na intake of less than 2 g/day (equivalent to <85 mmol/day), a salt equivalent intake of 5.1 g/day, and a K intake of at least 3.51 g/day (equivalent to ≥90 mmol/day) [16, 17], whereas the 2019 dietary guidelines of the Japanese Society of Hypertension (JSH) recommended a salt intake of less than 6 g/day (equivalent to Na  < 2.36 g/day [102.6 mmol/day]) [18].

Although a few multiethnic studies suggested a preferable urinary Na/K ratio cutoff range between 1‒2 molar ratios using standard dietary guidelines [8, 19, 20], a cutoff value exclusive to the Japanese population following the recently revised DRIs guidelines has not yet been investigated. Additionally, to estimate if the dietary intakes of salt (Na equivalent) and K matches the recommendations proposed by the DRIs guidelines, the excretion of these elements in urine could help in dietary goal assessment and adjusting dietary habits using the urinary Na/K ratio. The relevance of this knowledge will serve to update the guidelines for the optimal urinary Na/K ratio cutoff level and promote individualized dietary plans and the careful monitoring of urinary excretion. Therefore, we investigated the cutoff level of the urinary Na/K ratio in a middle-aged Japanese population from an international cooperative epidemiological study using high-quality data from two 24 h urine collections and four 24 h dietary recalls.

Point of View **Clinical relevance**

A urinary Na/K ratio cutoff level of approximately 2 may warrant simultaneously meeting the daily dietary goals for Na and K as recommended by the DRIs guidelines for the Japanese population.

**Future directions**

Population studies using a more practical spot urine method to confirm the proposed cutoff level of approximately 2 for the urinary Na/K ratio are needed.

**Considerations for the Asian population**

The proposed cutoff of approximately 2 for the urinary Na/K ratio is a feasible goal to simultaneously meet the recommended daily intake for both Na and K per the Japanese DRIs guidelines accommodating the habitual diet in Japan.

## Methods

### Study population

The participants were from the INTERnational study of MAcro- and micronutrients and blood Pressure (INTERMAP), an epidemiological investigation conducted between 1997 and 1999 aiming to clarify the role of macro- and micronutrient intakes on BP using high quality standardized data collection. This study surveyed 4680 randomly selected men and women aged 40‒59 years from four countries (China, Japan, the UK, and the US). Detailed methods are described elsewhere [21, 22]. Here, we analyzed the INTERMAP data of 1145 Japanese participants from four study centers: Sapporo, Toyama, Aito Town, and Wakayama. Each participant had four 24 h dietary recalls, two complete timed 24 h urine collections, and eight BP measurements from four visits to their local research center. Each of the local research centers provided institutional ethics committee approval, and all participants provided written informed consent. This observational study is registered as NCT00005271.

### Demographic characteristics, lifestyle data collection, and measurements

The demographic characteristics, lifestyle data and medical data were obtained using interviewer-assisted questionnaires, including height, weight (both measured at the first and third visits), alcohol intake, smoking status, sociodemographic characteristics, physical activity level, vitamin/mineral supplement use, medical history and medication use. Body mass index (BMI) was calculated as weight (kg) divided by height squared (m^2^). At each of the four visits, two systolic blood pressures (SBPs) and diastolic blood pressures (DBPs) were measured by trained staff using a random-zero sphygmomanometer with an adjustable cuff size on the right arm after the participant was seated for at least 5 min in a quiet room [21]. We used the average of the eight total BP measurements. Alcohol intake was recorded at the 1st and 3rd visits for the preceding 7 days, and a total of 14 days of alcohol consumption recall was obtained. The weekly average alcohol consumption in mL was calculated, and participants were grouped into 0 mL/week, 1‒299 mL/week, and ≥300 mL/week.

### Dietary recall data collection

The four 24 h dietary recalls were divided into two pairs of successive days, and the pairs had intervals of 3‒6 weeks. One of the days selected for dietary recall was on a day following a weekend. Dietary data were collected and tape-recorded by trained certified interviewers using the in-depth multipass 24 h recall method, recording all food, drinks and supplements consumed in the previous 24 h [22]. The study center’s nutritionist listened to and checked all the dietary recall tapes. Participants were excluded from the study if (1) they did not attend any of the four visits to their local research center, (2) the dietary data collected were considered unreliable, or (3) any of the 24 h dietary recall energy intakes were <500 kcal/day or >5000 kcal/day. Data collected from the 24 h dietary recall were used to calculate nutrient intake using the Japanese INTERMAP food table compiled by a Japanese nutritionist based on the Japanese Standard Food Tables 4th edition in cooperation with the Nutrition Coordination Center [23, 24]. The average of the four 24 h dietary recalls was used, the dietary Na and K intakes were calculated as mmol/day, and the dietary Na/K ratio was calculated as mmol/mmol.

### Urine collection

At the 1st and 3rd visits, after participants emptied their bladder at the study center and received 2 of the standard 1 L plastic jars with boric acid for preservation, an observer timed the start of 24 h urinary collection for participation, and participants returned the following day (visits 2 and 4) to complete the 24 h urinary collection. Participants were asked to repeat their urine collection when (1) they reported “more than few drops” of missing urine, (2) the collection time fell outside the range of 22‒26 h, or (3) the total volume of urine was <250 mL. Participants were excluded from the study if they did not submit two complete urine collections. Urine aliquots were frozen and air-freighted to the Central Laboratory (Leuven, Belgium) [21]. To ensure quality control, 10% of the urine samples were randomly split at the study centers and forwarded to the laboratory under a different ID number. Emission flame photometry was used to measure the concentrations of urinary Na and K, which were reported as mmol/day. The average of the two 24 h urinary collections was used, and the 24 h Na/K ratio was calculated from the 24 h Na and K urinary excretion values obtained.

### Statistical analysis

Continuous and categorical variables are presented as the mean ± standard deviation (SD) and numbers (%), respectively. Na and K concentrations were converted from mmol/day to g/day, calculated as g/day = [mmol/day x atomic weight (Na = 23.0, K = 39.1)]÷1000. The 24 h estimated dietary salt (NaCl) intake was calculated as g/day, enumerated as NaCl (g/day) = dietary Na intakex2.54. Pearson’s partial correlation coefficient, adjusted for sex and age, and Pearson’s correlation coefficient were used to examine the correlation between the 24 h dietary Na/K ratio and 24 h urinary Na/K ratio, the 24 h dietary Na/K ratio and dietary Na and K intake, and the 24 h urinary Na/K ratio and urinary Na and K excretion. The partial correlation coefficient was reported for the overall population, and Pearson’s correlation coefficient was reported and stratified by sex. The Bland‒Altman method was used to examine the bias of agreement between the 24 h urinary and dietary Na/K ratios.

The 24 h urinary K excretion is a good estimate of the dietary K intake; however, an average of 63‒77% of the dietary K intake is reflected in the urinary K excretion [24–28]. To approximate the urinary K excretion to dietary K intake, we calculated a population-specific correction coefficient derived from the INTERMAP Japanese population by dividing the population’s average of two 24 h urinary K excretions by the population’s average of four 24 h dietary K intakes: correction coefficient = 48.9÷71.5 = 0.684. We then divided the 24 h urinary K excretion by the correction coefficient to calculate the corrected urinary K excretion equivalent to the dietary K intake. The corrected urinary K excretion was used in all the following analyses. Based on the dietary intake standards recommended by the 2020 Japanese DRIs for each sex and the overall population (men: Na intake < 2.95 g/day [NaCl <  7.5 g/day] and K intake ≥ 3 g/day; women: Na intake < 2.56 g/day [NaCl < 6.5 g/day], and K intake ≥ 2.6 g/day), receiver operating characteristic (ROC) curves were used to explore the relationships between (1) the 24 h urinary excretion of Na and K and 24 h urinary Na/K ratio, (2) the 24 h dietary intake of Na and K and 24 h dietary Na/K ratio, and (3) the 24 h dietary intake of Na and K and 24 h urinary Na/K ratio. The area under the receiver operating characteristic curve (AUC) was examined for the explored relationships and cutoff level satisfying the Japanese DRIs guidelines by sex. The Na/K ratio cutoff level was reported as follows: (1) the distance to 0,1: closest cutoff to (0,1) perfect prediction [minimum distance to X = 0, Y = 1], ${{{{{{{\mathrm{d}}}}}}}} = \sqrt {\left( {1 - {{{{{{{\mathrm{sensitivity}}}}}}}}} \right)^2 + \left( {1 - {{{{{{{\mathrm{specificity}}}}}}}}} \right)^2}$; (2) sensitivity = specificity: cutoff point equates to near equal sensitivity and specificity; and (3) the Youden index: the maximum vertical line from the uninformative line, Youden index = sensitivity+specificity −1 [29, 30]. ROC curves and cutoff values were also examined following the dietary intake guidelines of the WHO and JSH [16–18]. To test the accuracy of predicting urinary and dietary Na and K by the Na/K ratio under the range of cutoff values of less than 2.0, 3.0 and 4.0 by the DRIs guidelines, sensitivity and specificity tests were performed.

For clinical implementation purposes, Japanese-specific guidance tables were created to visualize the estimated urinary Na/K ratio by the dietary K intake estimated from the corrected urinary K excretion and the dietary salt intake estimated from the 24 h urinary Na excretion based on the Japanese DRIs dietary goals. Both dietary K intake and salt intake are presented in g/day. The molar urinary Na/K ratio was presented as the range of the mean ± SD by subtracting the SD value from the mean to calculate the lower limit and adding the SD to the mean value to calculate the upper limit. Following the Japanese DRIs guidelines, dietary salt intake (g/day) was divided into four groups: < 5, 5‒7.5, 7.5‒12, and ≥12 for men, and < 5, 5–6.5, 6.5–10, and ≥10 for women. Dietary K intake from the corrected urinary K excretion (g/day) was divided into three groups: < 2, 2‒3, and ≥3 for men, and <1.8, 1.8‒2.6, and ≥2.6 for women. The cross-classified tables are presented as a heatmap by sex with dietary salt intake in rows and dietary K intake in columns, displayed in a color-shaded matrix with the color expressing the severity of the range of the mean urinary Na/K ratio as follows: ≥6 (red), 3‒6 (yellow), 2–3 (green), and <2 (blue). All analyses were performed using SAS software, version 9.4 (SAS Institute, Cary, NC).

## Results

A total of 1145 Japanese participants from the INTERMAP study were analyzed in this study. Table 1 shows the characteristics of the overall participants by sex (574 men and 571 women). Among the overall participants, the average age was 49.4 ± 5.3 years, and 6.4% were taking hypertension medications. The average 24 h estimated dietary salt intake was 11.8 ± 3.2 g/day, and the average 24 h dietary K intake was 71.5 ± 18.0 mmol/day. The average estimated 24 h urinary salt excretion was 11.6 ± 3.3 g/day, and the corrected 24 h urinary K excretion was 71.4 ± 19.8 mmol/day. The average molar ratios of 24 h dietary and urinary Na/K were 3.0 ± 0.7 and 4.3 ± 1.3, respectively. The mentioned characteristics were observed to be higher in men.

**Table 1:** Characteristics of study participants (INTERMAP Japan, 1997‒1999, 1145 men and women aged 40–59 years)

Characteristics	Overall (*N* = 1145)	Men (*N* = 574)	Women (*N* = 571)
Age, years	49.4 ± 5.3	49.5 ± 5.3	49.2 ± 5.3
Body Mass Index, kg/m^2^	23.4 ± 2.9	23.7 ± 2.7	23.2 ± 3.1
Systolic blood pressure, mmHg	117.3 ± 13.8	120.4 ± 12.9	114.1 ± 13.9
Diastolic blood pressure, mmHg	73.7 ± 10.3	76.8 ± 10.0	70.5 ± 9.6
Dietary recall			
24 h dietary Na, mmol/day	202.2 ± 55.6	221.7 ± 57.4	182.6 ± 46.2
24 h dietary salt, g/day	11.8 ± 3.2	13.0 ± 3.4	10.7 ± 2.7
24 h dietary K, mmol/day	71.5 ± 18.0	74.2 ± 18.5	68.7 ± 17.0
24 h dietary Na/K ratio	3.0 ± 0.7	3.1 ± 0.7	2.8 ± 0.6
Urine collection			
24 h urinary Na, mmol/day	198.3 ± 56.2	210.5 ± 56.6	186.0 ± 53.1
24 h estimated salt intake, g/day	11.6 ± 3.3	12.3 ± 3.3	10.9 ± 3.1
24 h urinary K, mmol/day	48.9 ± 13.6	49.2 ± 13.3	48.5 ± 13.9
24 h corrected urinary K, mmol/day^a^	71.4 ± 19.8	72.0 ± 19.5	70.9 ± 20.3
24 h urinary Na/K ratio	4.3 ± 1.3	4.5 ± 1.3	4.1 ± 1.2
Anti-hypertensive medication, *n* (%)	73 (6.4)	34 (5.9)	39 (6.8)
Alcohol (ethanol) consumption, *n* (%)			
0 mL/week	261 (22.8)	46 (8.0)	215 (37.7)
1‒299 mL/week	648 (56.6)	303 (52.8)	345 (60.4)
≥300 mL/week	236 (20.6)	225 (39.2)	11 (1.9)

Values are presented as mean±standard deviation unless indicated otherwise

*Na* sodium, *K* potassium

Urinary salt and dietary salt are estimated from urinary Na and dietary Na, respectively

^a^24 h corrected urinary K was calculated by dividing average 24 h urinary K by average 24 h dietary K intake; correction coefficient=48.9÷71.5 = 0.684, then dividing 24 h urinary K by the correction coefficient

Sex- and age-adjusted Pearson’s partial correlation coefficients for the 24 h urinary Na/K ratio with Na and K excretion were 0.52 and −0.49, respectively, while those with Na and K intake were 0.20 and −0.27, respectively (*P* < 0.001) (Supplementary Tables 1, 2, 3). Pearson’s correlation coefficients were examined by sex (Supplementary Tables 2 and 3), and the correlation coefficients for the 24 h urinary Na/K ratio with Na and K excretion were slightly higher for women. The bias between the 24 h dietary and urinary Na/K ratios using the Bland‒Altman method showed good agreement (bias 1.31) (Supplementary Fig. 1).

**Fig. 1:**
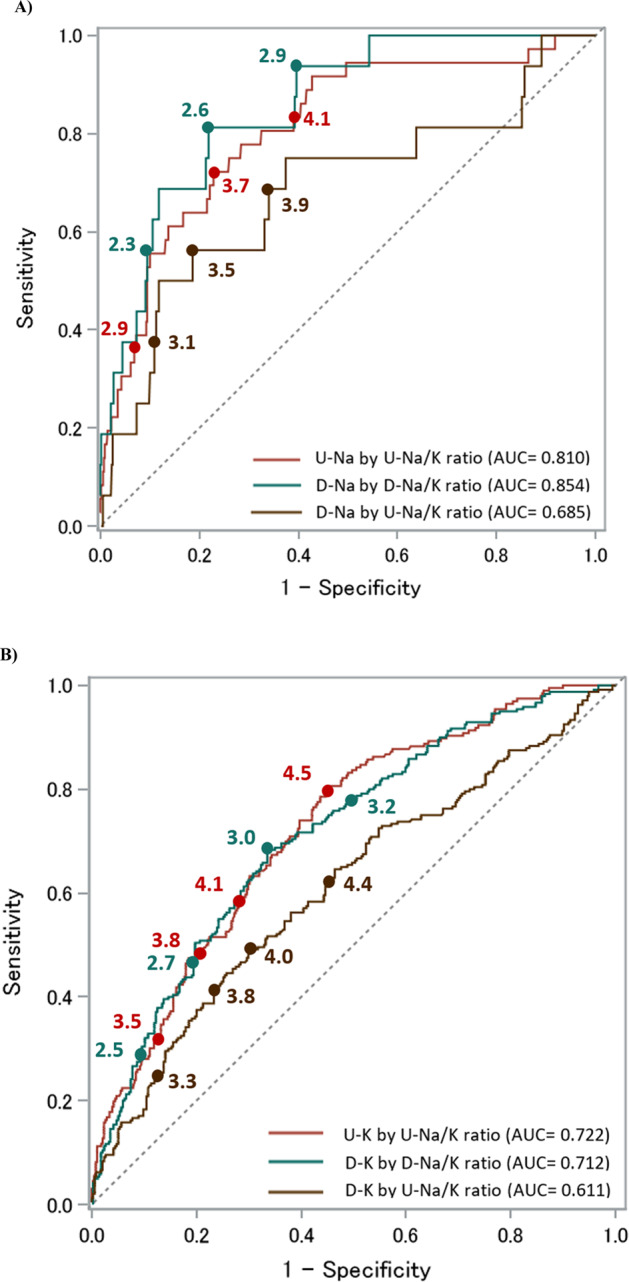
The area under the receiver operating characteristic curve (AUC) of the 24 h urinary and dietary Na/K ratio based on the DRIs recommendations for Na and K dietary intake for men (*N* = 574). **A** Predicting Na excretion and dietary intake by the 24 h urinary Na/K ratio and dietary Na/K ratio (DRI for men: Na < 2.95 g/day [128.3 mmol/day] or salt < 7.5 g/day) **B** Predicting K excretion and dietary intake by the 24 h urinary Na/K ratio and dietary Na/K ratio (DRI for men: K ≥ 3 g/day [77 mmol/day]). U-Na urinary sodium, D-Na dietary sodium, U-K urinary potassium, D-K dietary potassium, U-Na/K urinary Na/K, D-Na/K dietary Na/K

**Fig. 2:**
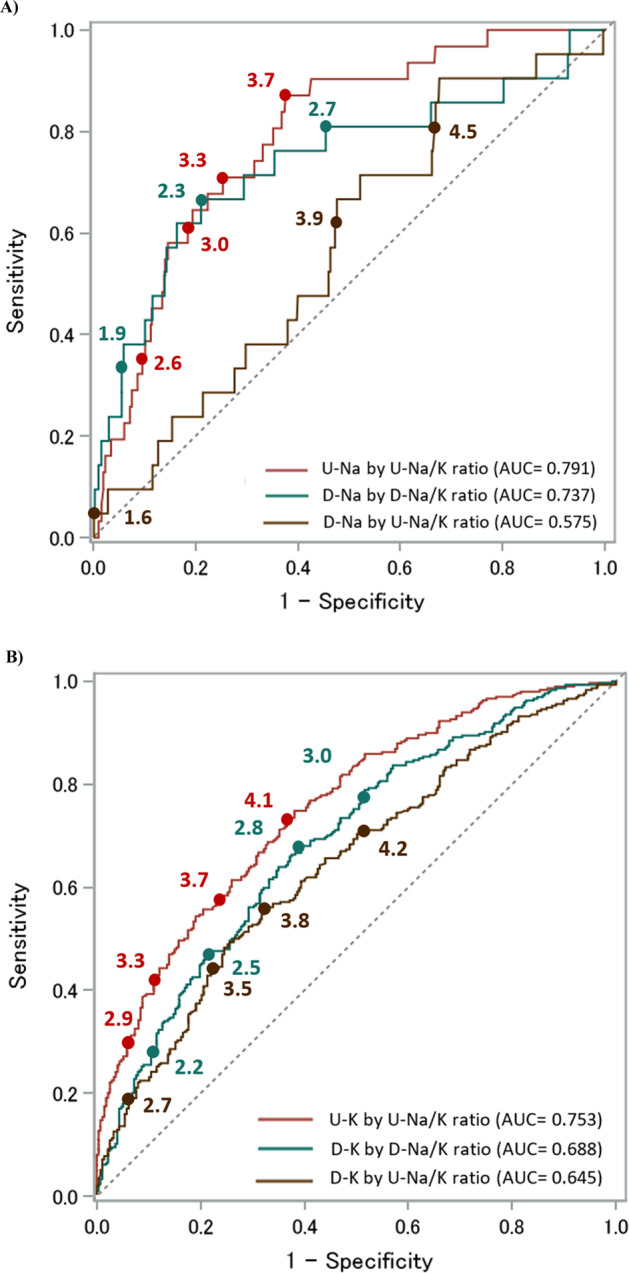
The area under the receiver operating characteristic curve (AUC) of the 24 h urinary and dietary Na/K ratio based on the DRIs recommendations for Na and K dietary intake for women (*N* = 571). **A** Predicting Na excretion and dietary intake by the 24 h urinary Na/K ratio and dietary Na/K ratio (DRI for women: Na <2.56 g/day [111.3 mmol/day] or salt <6.5 g/day). **B** Predicting K excretion and dietary intake by the 24 h urinary Na/K ratio and dietary Na/K ratio (DRI for women: K ≥ 2.6 g/day [67 mmol/day]). U-Na urinary sodium, D-Na dietary sodium, U-K urinary potassium, D-K dietary potassium, U-Na/K urinary Na/K, D-Na/K dietary Na/K

**Fig. 3:**
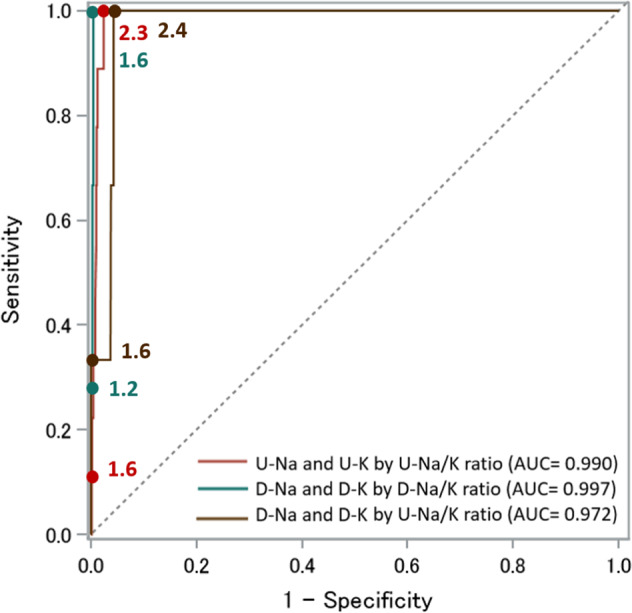
The area under the receiver operating characteristic curve (AUC) of the 24 h urinary and dietary Na/K ratio based on the DRIs recommendations for Na and K dietary intake by sex in the overall INTERMAP population (*N* = 1145). Predicting Na and K excretion and dietary intake by the 24 h urinary Na/K ratio and dietary Na/K ratio (Na for men: <7.5 g/day (Na < 2.95 g/day [128.3 mmol/day]) and for women: <6.5 g/day (Na < 2.56 g/day [111.3 mmol/day]) and (K for men: K ≥ 3 g/day [77 mmol/day] and for women: K ≥ 2.6 g/day [67 mmol/day]). U-Na urinary sodium, D-Na dietary sodium, U-K urinary potassium, D-K dietary potassium, U-Na/K urinary Na/K, D-Na/K dietary Na/K

Figures 1 and 2 show the ROC curves of the 24 h urinary and dietary Na/K ratios based on the DRIs-recommended dietary Na and K intake for men (Na < 2.95 g/day [128.3 mmol/day], K ≥ 3 g/day [77 mmol/day]) and women (Na < 2.56 g/day [111.3 mmol/day], K ≥ 2.6 g/day [67 mmol/day]), respectively. Table 2 shows the cutoff level for predicting urinary and dietary Na and K by 24 h urinary and dietary Na/K ratios following the DRIs dietary goals by sex. Using the three determining methods of the optimal cutoff level, most of the 24 h urinary Na/K ratio values predicting Na and K excretion separately following the DRIs dietary goals by sex fell between 3 and 4. The AUC for the relationship between the 24 h urinary Na/K ratio and dietary Na and K intakes was the lowest compared with those for the 24 h urinary Na/K ratio and urinary Na and K excretion and the 24 h dietary Na/K ratio and dietary Na and K intakes. The ROC curves and AUCs of the 24 h urinary and dietary Na/K ratios based on the DRIs recommendations for both dietary Na and K intakes together in the overall INTERMAP population showed higher predictivity than each element alone (Fig. 3). The cutoff of approximately 2 for the urinary Na/K ratio showed better predictions of estimated Na and K intakes by the guidelines’ recommended levels.

**Table 2:** Cutoff value of Na/K ratio for predicting dietary goals of Na and K intake in DRI guidelines using ROC analysis (574 men and 571 women)

					Na/K ratio cut-off level by ROC^a^			
	DRI dietary goal reference value		No. of participants	No. fitting dietary goals^b^	Distance to 0,1	Sensit = Specif	Youden index	AUC
DRIs_Na Men	7.5 g/day	U-Na by U-Na/K ratio	574	36	3.7	3.8	3.7	0.810
		D-Na by D-Na/K ratio		16	2.6	2.6	2.6	0.854
		D-Na by U-Na/K ratio		16	4.0	3.9	3.2	0.685
DRIs_K Men	3 g/day	U-K by U-Na/K ratio	574	196	4.2	4.2	4.6	0.722
		D-K by D-Na/K ratio		240	3.0	3.0	3.0	0.712
		D-K by U-Na/K ratio		240	4.2	4.3	4.0	0.611
DRIs_Na Women	6.5 g/day	U-Na by U-Na/K ratio	571	31	3.3	3.4	3.7	0.791
		D-Na by D-Na/K ratio		21	2.3	2.4	2.3	0.737
		D-Na by U-Na/K ratio		21	3.9	3.9	4.6	0.575
DRIs_K Women	2.6 g/day	U-K by U-Na/K ratio	571	298	3.6	3.5	3.6	0.753
		D-K by D-Na/K ratio		294	2.8	2.8	2.8	0.688
		D-K by U-Na/K ratio		294	3.8	4.0	3.8	0.645

*Sensit* sensitivity, *Specif* specificity, *AUC* area under curve, *DRIs_Na* Dietary Reference Intakes dietary goal for sodium, *DRIs_K* Dietary Reference Intakes dietary goal for potassium, *U-Na* urinary sodium, *D-Na* dietary sodium, *U-K* urinary potassium, *D-K* dietary potassium, *U-Na/K* urinary Na/K, *D-Na/K* dietary Na/K

^a^Na/K ratio cut-off level by ROC analysis was reported as (1) Distance to 0,1: closest cut-off to (0,1) perfect prediction [minimum distance to X = 0, Y = 1] $d = \sqrt {\left( {1 - sensitivity} \right)^2 + \left( {1 - specificity} \right)^2}$, (2) Sensit = Specif: cut-off point equates to near equal sensitivity and specificity, and (3) Youden index: maximum vertical line from uninformative line, Youden index=sensitivity + specificity −1

^b^Fitting dietary goals defined as actual number of participants out of total by gender that fit the criteria of Na and K dietary intake respectively according to DRIs as for men (Na < 2.95 g/day [128.3 mmol/day]) and less than 6.5 g/day for women (Na < 2.56 g/day [111.3 mmol/day]), and K intake of at least 3 g/day for men (77 mmol/day) and at least 2.6 g/day for women (67 mmol/day)

The AUCs and cutoff levels were also examined for the 24 h urinary Na/K ratio based on the recommended guidelines of the WHO dietary goals for Na and K and the JSH dietary goal for Na (Supplementary Tables 4 and 5 and Supplementary Figs 2 and 3). The cutoff threshold for the 24 h urinary Na/K ratio predicting Na and K separately under these two guidelines was similar to the DRIs threshold ranging between 3 and 4.

Additionally, in the sensitivity and specificity analysis of the 24 h urinary and dietary Na and K predicted by the Na/K ratio, the cutoff value between 3 and 4 seemed to show better predictions for each element separately (Supplementary Tables 6, 7).

**Fig. 4:**
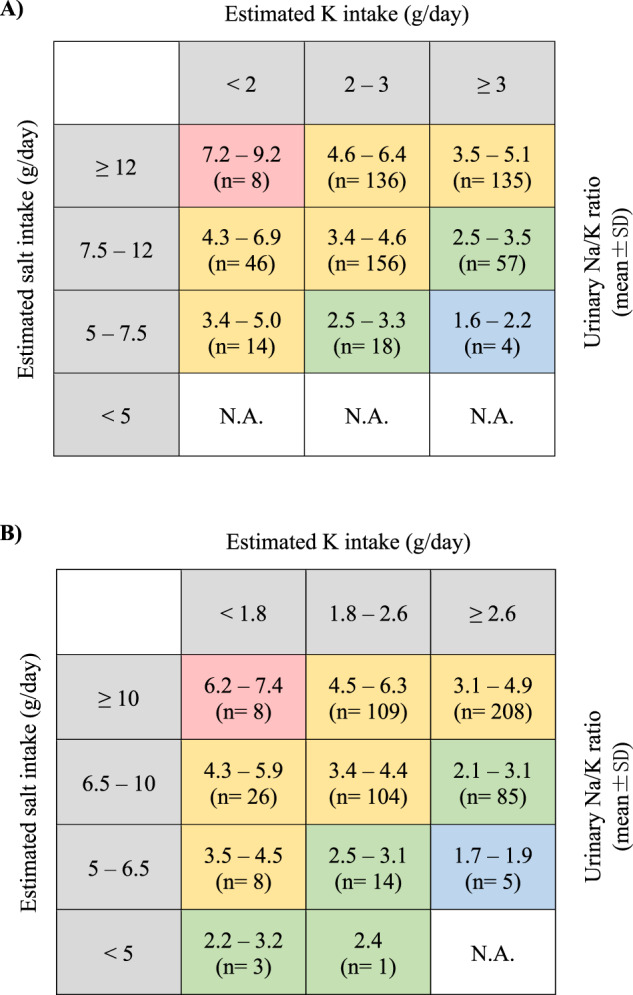
Range (means ± standard deviations) and number of participants in each category of the 24 h urinary Na/K ratio, cross classified by estimated salt and K intake by sex. **A** In men (*N* = 574), and **B** in women (*N* = 571). Color-shaded matrix guide of the range of the mean urinary Na/K ratio: red, urinary Na/K ratio ≥6, yellow, urinary Na/K ratio of 3–6, green, urinary Na/K ratio of 2-3, blue, urinary Na/K ratio <2. The DRIs dietary goal of salt intake of <7.5 g/day (Na < 2.95 g/day [128.3 mmol/day]) for men and <6.5 g/day (Na < 2.56 g/day [111.3 mmol/day]) for women. The daily K intake goal is ≥3 g/day for men (77 mmol/day) and ≥2.6 g/day for women (67 mmol/day). N.A. not applicable, SD standard deviation, K potassium

The Japanese sex-specific guidance tables for the 24 h urinary Na/K ratio under the DRIs dietary goals for Na intake (presented as salt intake estimated from urinary Na excretion) and K intake (presented as dietary K intake in g/day estimated from the corrected urinary K excretion) showed the optimal 24 h urinary Na/K ratio range between 1.6 and 2.2 for men when the dietary K intake was greater than or equal to 3 g/day and the dietary salt intake was less than 7.5 g/day (the number of men meeting both dietary intake recommendations was 4). For women, the optimal 24 h urinary Na/K ratio range was between 1.7 and 1.9 when the dietary K intake was greater than or equal to 2.6 g/day and the dietary salt intake was less than 6.5 g/day (the number of women meeting both dietary intake recommendations was 5) (Fig. 4).

## Discussion

In this study, we investigated the cutoff level of the 24 h urinary Na/K ratio in middle-aged Japanese men and women based on three dietary guidelines, mainly the Japanese DRIs. Overall, the 24 h urinary Na/K ratio showed better predictions of Na and K than the 24 h dietary Na/K ratio. The ROC curve of the 24 h urinary Na/K ratio at a cutoff of approximately 2 predicted combined Na and K dietary intake based on the recommended DRIs guidelines by sex. Furthermore, the range of urinary Na/K ratios to meeting both Na and K dietary goals simultaneously shown in the cross-classified tables was 1.6‒2.2 for men and 1.7‒1.9 for women. Advocating for a urinary Na/K ratio of approximately 2 would be desirable to achieve the goals for both Na and K intake recommended by the Japanese DRIs, which takes into account the habitual diet of the Japanese population.

It is widely accepted that 24 h urine collection is the most reliable measure for assessing the dietary intake of Na and K in clinical and epidemiological studies. Previous reports have shown an association between the urinary 24 h Na/K ratio and BP [7, 8, 13, 19]. Although the estimation of the dietary intake of these nutrients from 24 h urine collection is valid, it is inconvenient and involves considerable burden for participants in epidemiological studies and patients in clinical settings. Existing evidence shows, with appropriate bias correction, that the mean of 6 random daytime spot urine samples is a suitable estimate of an individual’s Na/K ratio, substituting for 2-day 24 h urine collection. The Pearson correlation coefficient of the means of the 24 h urine and spot urine Na/K ratio was *r* = 0.96 across different INTERSALT populations [3, 4]. In this study, we used the average of 2 repeated 24 h urine collections to estimate the Na/K ratio, which is the first study in the Japanese population using high standard repeated measurements of 24 h urine collection. Even though comparable high-quality studies are limited due to its high burden and low feasibility and more population-based evidence is needed to solidify the findings in this paper, the use of spot urine to estimate the Na/K ratio is an affordable, practical substitute and is comparable to the 24 h urinary Na/K ratio.

Both the 24 h urinary and dietary Na/K ratios predicted urinary and dietary Na and K under the sex-specific DRIs dietary goals, and overall, the urinary Na/K ratio showed slightly better predictions than the dietary Na/K ratio. A paper from the INTERSALT study used casual and 24 h urinary Na/K ratios to predict Na intake following the WHO dietary goal of Na < 85 mmol/day (Na <  2 g/day [salt intake < 5 g/day] [20]. They showed that the 24 h urinary Na/K ratio was a better predictor than casual urine samples, with an AUC similar to our findings (24 h urine Na/K ratio AUC=0.84). In addition, they found that the 24 h urinary Na/K ratio could predict Na intake under the WHO dietary goal with a sensitivity over 90% when the Na/K ratio was less than 1. In the current study, the cutoff level of urinary 24 h Na/K predicting Na intake following the sex-specific DRIs dietary goal was between 3 and 4 for Na and K separately; however, the cutoff level of approximately 2 for both elements simultaneously was deemed to be appropriate. Nevertheless, the gap between the two studies could be due to the lower dietary goal of the WHO and the relatively lower K intake in Japanese individuals.

An additional note seen from the sensitivity and specificity results of the current paper reveals that participants with lower Na dietary intake also show a tendency to have lower K intake. In the dietary recommendations, it is important to emphasize decreasing Na intake and increasing K intake to balance out the estimated Na/K ratio and reach the proposed threshold in this paper.

The sex-specific cross-classified tables of salt and K intakes showed a desirable range of the mean 24 h urinary Na/K ratio to meet the DRIs dietary goals for both Na and K with a range of 1.6‒2.2 for men and 1.7‒1.9 for women. It is important to mention that the total number of participants meeting both dietary goals recommended by the DRIs was only 9, which was <1% of the 1145 participants. Nevertheless, this is the first study to try and establish sex-specific dietary goals for salt and K intakes evaluated by the urinary Na/K ratio. The suggested optimal range of the urinary Na/K ratio is relevant to the proposed level of urinary Na/K cutoff value of approximately 2 [8, 19, 20]. Compared to international dietary guidelines for the two elements, the Japanese dietary guideline recommendations are considerably high for Na (salt equivalent) and low for K. One of the reasons is to accommodate the special dietary habits and sources of intake for Na and K in the Japanese diet. Additionally, although the international guidelines of strict dietary intake to reach a Na/K ratio of 1 could be difficult to achieve in some populations and would require drastic changes in dietary habits nationwide, it is important to advocate for the proper threshold considering the applicability of the proposed level per population; we conclude that a cutoff level of approximately 2 used for our Japanese participants may help them achieve the recommended dietary goal for both Na and K simultaneously and it is not far from the internally proposed threshold and guidelines. Several papers emphasized the importance of setting a practical cutoff level of the Na/K ratio, which is appropriate for the population’s dietary habits and is applicable for implementation in medical practice to follow the diet and health of the population [20, 31, 32]. The implications of this study’s findings of the cutoff value for the urinary Na/K ratio might instigate further population-based studies to help establish an acceptable and practical guidelines for estimating dietary salt and K intake using the urinary Na/K ratio in a middle-aged Japanese population.

This study has some limitations. First, the study participants were from the Japanese population of middle-aged individuals aged 40 to 59 years. It is difficult to apply the findings to other countries and younger or older age groups. Second, in the sex-specific cross-classified table, the number of participants meeting the goals for both salt and K intake following the Japanese DRIs guidelines was low (4 men and 5 women), which might be due to the low amount of raw and leafy vegetables as a source of K in the Japanese diet. Third, 24 h urine collection is the gold standard for measuring Na and K excretion; nonetheless, it is an impractical measure for monitoring population intake of these elements. A more feasible measure, such as repeated spot urine samples, might be a future approach to confirm the cutoff level of the urinary Na/K ratio. Finally, the data were collected more than 20 years ago, which may affect the relationship of dietary and urinary Na and K values considering the changes in health habits, medications, and dietary intake sources of these elements, among other factors. However, we believe the application of our findings from the matrix tables of the urinary Na/K ratio by salt and K intake are important, and further studies are needed to investigate similar concepts with recent data. There are noteworthy strengths to this study. We used high-quality standardized data in a general population from two 24 h urine collections, which is the gold standard for assessing salt intake. Finally, the average of two urinary collections and four dietary recalls were used, which would be a representative measure of the actual habitual intake compared to a single measure.

### Perspective of Asia

The WHO recommends strict daily intake goals for Na (<2 g/day) and K ( > 3.51 g/day), which yields Na/K ratio of ≤1.0 measured by urinary excretion [16, 17]. However, this study concluded that a urinary Na/K ratio cutoff level of approximately 2 was feasible for simultaneously meeting the daily goals for both Na and K in the Japanese DRIs guidelines. Following the local recommendations was deemed appropriate to accommodate the dietary supply and habitual consumption of food by the Japanese population.

In Japan and other Asian countries, it is important to use the proposed Na/K ratio as an index to monitor the dietary intake of these essential health elements, especially in Asia, where the diet is composed of high salt and low potassium intake, leading to an imbalance of these elements by daily recommendations.

In conclusion, in the general middle-aged Japanese population, a 24 h urinary Na/K ratio cutoff level of approximately 2 would be desirable to simultaneously achieve dietary goal recommendations for both Na and K and may help the population meet the recommended dietary goals for Na and K intake by the Japanese DRIs. An achievable level of the 24 h urinary Na/K ratio could serve as a better approach to help advocate for lowering Na intake and increasing K intake in the Japanese population.

## Supplementary information

Supplementary Tables

Supplementary Figures
