# Supplementary material for: Investigation of the urinary sodium-to-potassium ratio target level based on the recommended dietary intake goals for the Japanese population: The INTERMAP Japan
Source: Hypertens Res. 2022 Nov 8;45(12):1850–60. doi: 10.1038/s41440-022-01007-x (PMC9659487; doi:10.1038/s41440-022-01007-x)
Supplement: Supplementary file 1 — Supplementary Tables [file 41440_2022_1007_MOESM1_ESM.docx]

**Supplementary Table 1. Pearson’s partial correlation coefficients for 24 h urinary excretion and dietary intake of Na, K and Na/K ratio adjusting for sex-age (N = 1145)**

|  |  |  | 24 h urinary Na excretion | 24 h urinary  K excretion | 24 h dietary Na/K ratio | 24 h dietary Na intake | 24 h dietary K  intake |
| --- | --- | --- | --- | --- | --- | --- | --- |
| 24 h urinary Na/K ratio | | | 0.524* | - 0.493* | 0.530* | 0.198* | - 0.265* |
| 24 h urinary Na excretion | | | ‒ | 0.414* | 0.213* | 0.455* | 0.258* |
| 24 h urinary K excretion | | |  | ‒ | - 0.356* | 0.223* | 0.560* |
| 24 h dietary Na/K ratio | | |  |  | ‒ | 0.427* | - 0.405* |
| 24 h dietary Na intake | | |  |  |  | ‒ | 0.607* |
| 24 h dietary K intake | | |  |  |  |  | ‒ |

Units of Na and K excretion and intake are in mmol/day

* *P* < 0.001

**Supplementary Table 2. Pearson’s correlation coefficients for 24 h urinary excretion and dietary intake of Na, K and Na/K ratio for men (N = 574)**

|  |  |  | 24 hr urinary Na excretion | 24 hr urinary  K excretion | 24 hr dietary Na/K ratio | 24 hr dietary Na intake | 24 hr dietary  K  intake |
| --- | --- | --- | --- | --- | --- | --- | --- |
| 24 hr urinary Na/K ratio | | | 0.504* | - 0.469* | 0.498* | 0.189* | - 0.229* |
| 24 hr urinary Na excretion | | | ‒ | 0.452* | 0.192* | 0.485* | 0.299* |
| 24 hr urinary K excretion | | |  | ‒ | - 0.329* | 0.261* | 0.549* |
| 24 hr dietary Na/K ratio | | |  |  | ‒ | 0.410* | - 0.406* |
| 24 hr dietary Na intake | | |  |  |  | ‒ | 0.623* |
| 24 hr-dietary K intake | | |  |  |  |  | ‒ |

Units of Na and K excretion and intake are in mmol/day

* *P* < 0.001

**Supplementary Table 3. Pearson’s correlation coefficients for 24 h urinary excretion and dietary intake of Na, K and Na/K ratio for women (N = 571)**

|  |  |  | 24 h urinary Na excretion | 24 h urinary  K excretion | 24 h dietary Na/K ratio | 24 h dietary Na intake | 24 h dietary  K  intake |
| --- | --- | --- | --- | --- | --- | --- | --- |
| 24 h urinary Na/K ratio | | | 0.543* | - 0.510* | 0.564* | 0.213* | - 0.296* |
| 24 h urinary Na excretion | | | ‒ | 0.388* | 0.219* | 0.423* | 0.232* |
| 24 h urinary K excretion | | |  | ‒ | - 0.398* | 0.191* | 0.586* |
| 24 h dietary Na/K ratio | | |  |  | ‒ | 0.436* | - 0.415* |
| 24 h dietary Na intake | | |  |  |  | ‒ | 0.591* |
| 24 h dietary K intake | | |  |  |  |  | ‒ |

Units of Na and K excretion and intake are in mmol/day

* *P* < 0.001

**Supplementary Table 4. Number and percentage of participants fitting the dietary goals of Na and K in three dietary guidelines (N = 1145, men = 574, women = 571)**

| Guidelines | Nutrient | Dietary | Urinary |
| --- | --- | --- | --- |
| WHO | Na | 4 (0.4) | 4 (0.4) |
|  | cU-K | 157 (13.7) | 200 (17.5) |
| DRIs_men | Na | 16 (2.8) | 36 (6.3) |
|  | cU-K | 240 (41.8) | 196 (34.2) |
| DRIs_women | Na | 21 (3.7) | 31 (5.4) |
|  | cU-K | 294 (51.5) | 298 (52.2) |
| JSH | Na | 15 (1.3) | 18 (1.6) |

Values are presented as number (%). WHO, World Health Organization; DRIs_men, Dietary Reference Intakes for men; DRIs_women: Dietary Reference Intakes for women; JSH, Japanese Society of Hypertension; Na, sodium; K, potassium.

cU-K: corrected 24-h urinary K excretion

World Health Organization dietary goal: Na < 2 g/day [85 mmol/day] or salt < 2 g/day, K ≥ 3.51 [90 mmol/day]. Dietary Reference Intakes for men: Na < 2.95 g/day [128.3mmol/day] or salt < 7.5 g/day), K ≥ 3 g/day [77 mmol/day]. Dietary Reference Intakes for women: Na < 2.56 g/day [111.3 mmol/day] or salt < 6.5 g/day), K ≥ 2.6 g/day [67 mmol/day]. Japanese Society of Hypertension dietary goal for Na < 2.36 g/day [102.6 mmol/day] or salt < 6 g/day.

**Supplementary Table 5. Cut-off threshold for predicting dietary goals of Na and K intake in WHO and JSH guidelines using ROC analysis (N = 1145)**

|  | |  |  |  | Na/K ratio cutoff level by ROC | | |  |
| --- | --- | --- | --- | --- | --- | --- | --- | --- |
|  |  | | No. of participants | No. fitting guideline† | Distance to 0,1 | Sensit = Specif | Youden index | AUC |
| WHO_Na | U-Na by U-Na/K ratio | | 1145 | 4 | 3.2 | 3.2 | 3.2 | 0.909 |
|  | D-Na by D-Na/K ratio | |  | 4 | 2.2 | 2.5 | 2.2 | 0.860 |
|  | D-Na by U-Na/K ratio | |  | 4 | 3.9 | 3.9 | 2.7 | 0.704 |
| WHO_K | cU-K by U-Na/K ratio | | 1145 | 200 | 3.8 | 3.8 | 3.8 | 0.755 |
|  | D-K by D-Na/K ratio | |  | 157 | 2.8 | 2.8 | 2.9 | 0.681 |
|  | D-K by U-Na/K ratio | |  | 157 | 4.1 | 3.9 | 4.1 | 0.645 |
| JSH_Na | U-Na by U-Na/K ratio | | 1145 | 18 | 2.9 | 3.1 | 2.9 | 0.898 |
|  | D-Na by D-Na/K ratio | |  | 15 | 2.2 | 2.4 | 2.2 | 0.859 |
|  | D-Na by U-Na/K ratio | |  | 15 | 3.9 | 3.9 | 4.0 | 0.681 |

Sensit, sensitivity; Specif, specificity; AUC, area under curve; U-Na, urinary sodium; D-Na, dietary sodium; cU-K, corrected urinary potassium; D-K, dietary potassium; U-Na/K, urinary Na/K; D-Na/K, dietary Na/K; WHO_Na, World Health Organization dietary goal for sodium; WHO_K: World Health Organization dietary goal for potassium; JSH_Na, Japanese Society of Hypertension dietary goal for sodium.

†Fitting dietary goals defined as actual number of participants out of the total number that fit the criteria of Na and K dietary intake goal by WHO respectively as Na intake of less than 2 g/day (equivalent to < 85 mmol/day) and at least 3.51 g/day of K intake (equivalent to ≥ 90 mmol/day). Similarly of fitting dietary goal defined as actual number of participants out of the total number that fit JSH recommended salt intake to less than 6 g/day (equivalent to Na < 2.36 g/day [102.6 mmol/day]).

**Supplementary Table 6. Sensitivity and specificity of urinary and dietary Na predicted by Na/K ratio for cutoff values less than 2.0, 3.0 and 4.0 by DRIs guidelines (N= 1145)**

|  |  | Na/K ratio cutoff value | | |
| --- | --- | --- | --- | --- |
|  |  | < 2.0 (95% CI) | < 3.0 (95% CI) | < 4.0 (95% CI) |
| Sensitivity | U-Na by U-Na/K ratio | 11.9% (4.2 ‒ 19.7) | 47.8% (35.8 ‒ 59.7) | 85.1% (76.5 ‒ 93.6) |
|  | D-Na by D-Na/K ratio | 29.7% (15.0 ‒ 44.5) | 86.5% (75.5 ‒ 97.5) | 100% |
|  | D-Na by U-Na/K ratio | 8.1% (0.0 ‒ 16.9) | 24.3% (10.5 ‒ 38.2) | 70.3% (55.5 ‒ 85.0) |
| Specificity | U-Na by U-Na/K ratio | 98.6% (97.9 ‒ 99.3) | 86.9% (84.9 ‒ 88.9) | 56.7% (53.7 ‒ 59.6) |
|  | D-Na by D-Na/K ratio | 95.3% (94.1 ‒ 96.6) | 44.0% (41.0 ‒ 46.9) | 7.2% (5.7 ‒ 8.7) |
|  | D-Na by U-Na/K ratio | 98.2% (97.4 ‒ 99.0) | 85.2% (83.1 ‒ 87.3) | 55.1% (52.1 ‒ 58.0) |

The DRIs daily dietary goal of salt intake for men < 7.5 g/day (Na < 2.95 g/day [128.3 mmol/day]) and for women < 6.5 g/day (Na < 2.56 g/day [111.3 mmol/day]).

U-Na: urinary sodium, D-Na: dietary sodium, U-Na/K: urinary Na/K, D-Na/K: dietary Na/K.

**Supplementary Table 7. Sensitivity and specificity of urinary and dietary K predicted by Na/K ratio for cutoff values less than 2.0, 3.0 and 4.0 by DRIs guidelines (N= 1145)**

|  |  | Na/K ratio cutoff value | | |
| --- | --- | --- | --- | --- |
|  |  | < 2.0 (95% CI) | < 3.0 (95% CI) | < 4.0 (95% CI) |
| Sensitivity | cU-K by U-Na/K ratio | 4.7% (2.8 ‒ 6.5) | 27.5% (23.6 ‒ 31.5) | 65.0% (60.8 ‒ 69.2) |
|  | D-K by D-Na/K ratio | 9.4% (6.9 ‒ 11.8) | 74.2% (70.4 ‒ 77.9) | 97.8% (96.5 ‒ 99.0) |
|  | D-K by U-Na/K ratio | 3.6% (2.0 ‒ 5.1) | 21.7% (18.2 ‒ 25.2) | 56.4% (52.2 ‒ 60.6) |
| Specificity | cU-K by U-Na/K ratio | 100% | 94.3% (92.5 ‒ 96.1) | 68.8% (65.3 ‒ 72.4) |
|  | D-K by D-Na/K ratio | 97.9% (96.7 ‒ 99.0) | 57.9% (54.0 ‒ 61.9) | 11.1% (8.6 ‒ 13.6) |
|  | D-K by U-Na/K ratio | 99.4% (98.7 ‒ 100) | 90.7% (88.4 ‒ 93.0) | 62.5% (58.7 ‒ 66.3) |

The DRIs daily dietary goal of K intake is ≥ 3 g/day for men (77 mmol/day) and ≥ 2.6 g/day for women (67 mmol/day).

cU-K: corrected urinary potassium, D-K: dietary potassium, U-Na/K: urinary Na/K, D-Na/K: dietary Na/K.
