# Supplementary material for: Investigation of the urinary sodium-to-potassium ratio target level based on the recommended dietary intake goals for the Japanese population: The INTERMAP Japan
Source: Hypertens Res. 2022 Nov 8;45(12):1850–60. doi: 10.1038/s41440-022-01007-x (PMC9659487; doi:10.1038/s41440-022-01007-x)

**Figure Legends**

**Supplementary Fig. 1.** Bland-Altman plot of 24 h dietary Na/K ratio vs 24 h urinary Na/K ratio (n=1145 participants). Bias between 24 h dietary vs 24 h urinary Na/K ratio was 1.31.

**Supplementary Fig. 2.** The area under the receiver operating characteristic curve (AUC) of the 24 h urinary and dietary Na/K ratio based on the recommended WHO Na and K dietary intake (N = 1145). A) Predicting Na excretion and dietary intake by 24 h urinary Na/K ratio and dietary Na/K ratio (WHO: Na < 2 g/day [85 mmol/day]) B) Predicting K excretion and dietary intake by 24 h urinary Na/K ratio and dietary Na/K ratio (WHO: K ≥ 3.51 [90 mmol/day]). U-Na: urinary sodium, D-Na: dietary sodium, U-K: urinary potassium, D-K: dietary potassium, U-Na/K: urinary Na/K, D-Na/K: dietary Na/K.

**Supplementary Fig. 3.** The area under the receiver operating characteristic curve (AUC) of the 24 h urinary and dietary Na/K ratio based on the recommended JSH Na dietary intake (N = 1145). Predicting Na excretion and dietary intake by 24 h urinary Na/K ratio and dietary Na/K ratio (JSH: Na < 2.36 g/day [102.6 mmol/day]). U-Na: urinary sodium, D-Na: dietary sodium, U-Na/K: urinary Na/K, D-Na/K: dietary Na/K.

**Supplementary Fig. 1**


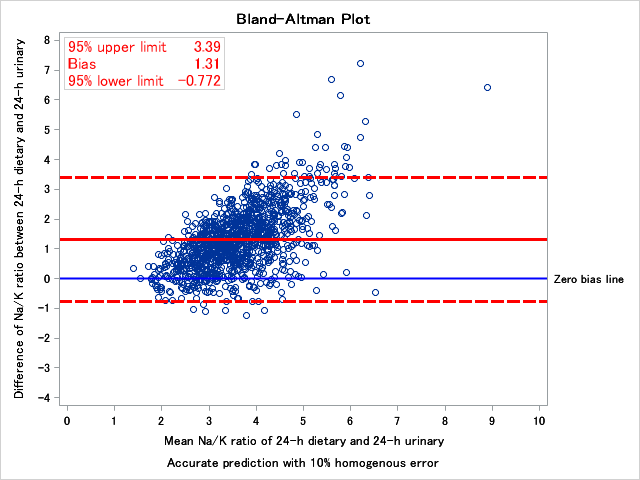


**Supplementary Fig. 2**


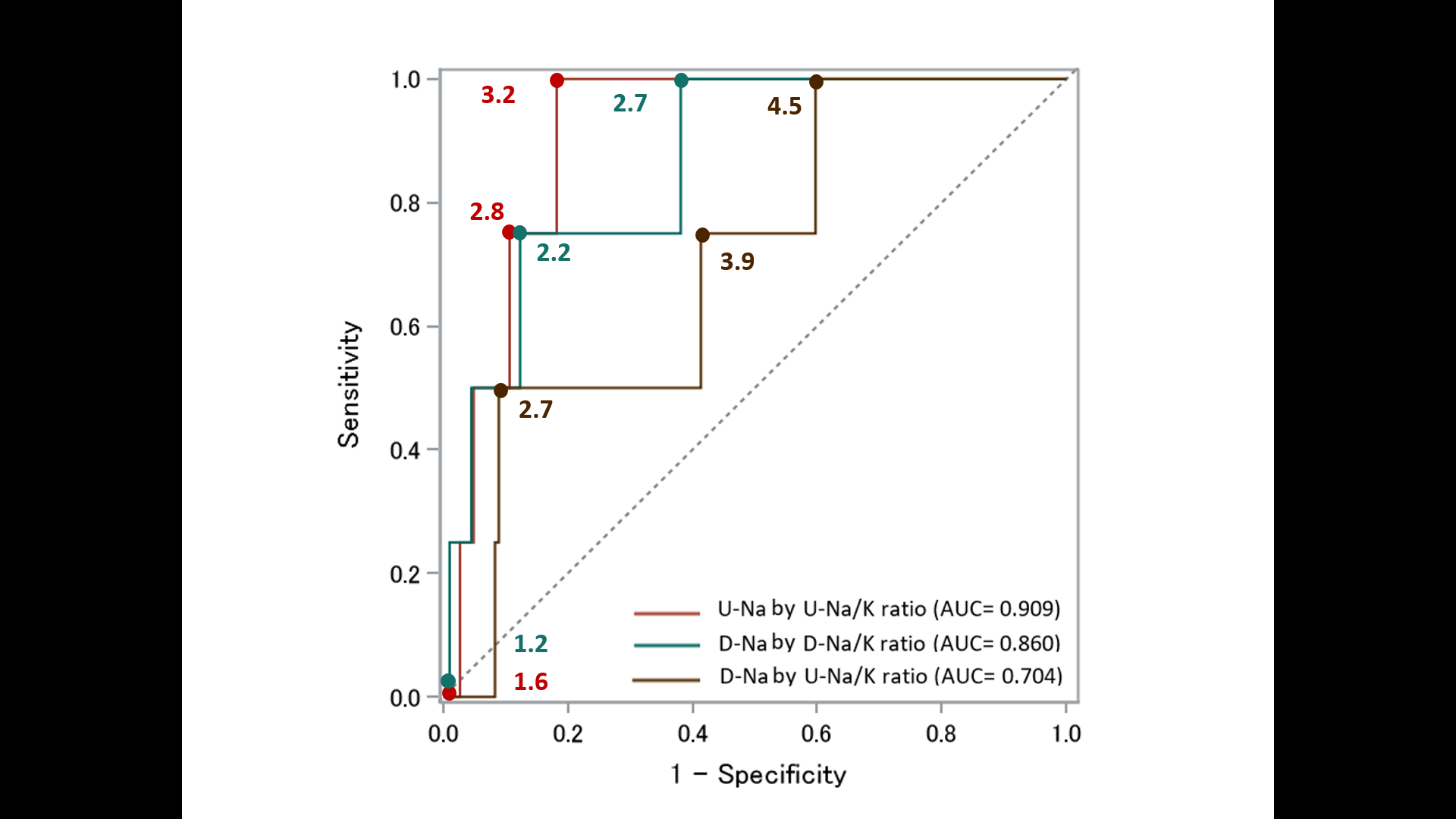
**A)**

**B)**


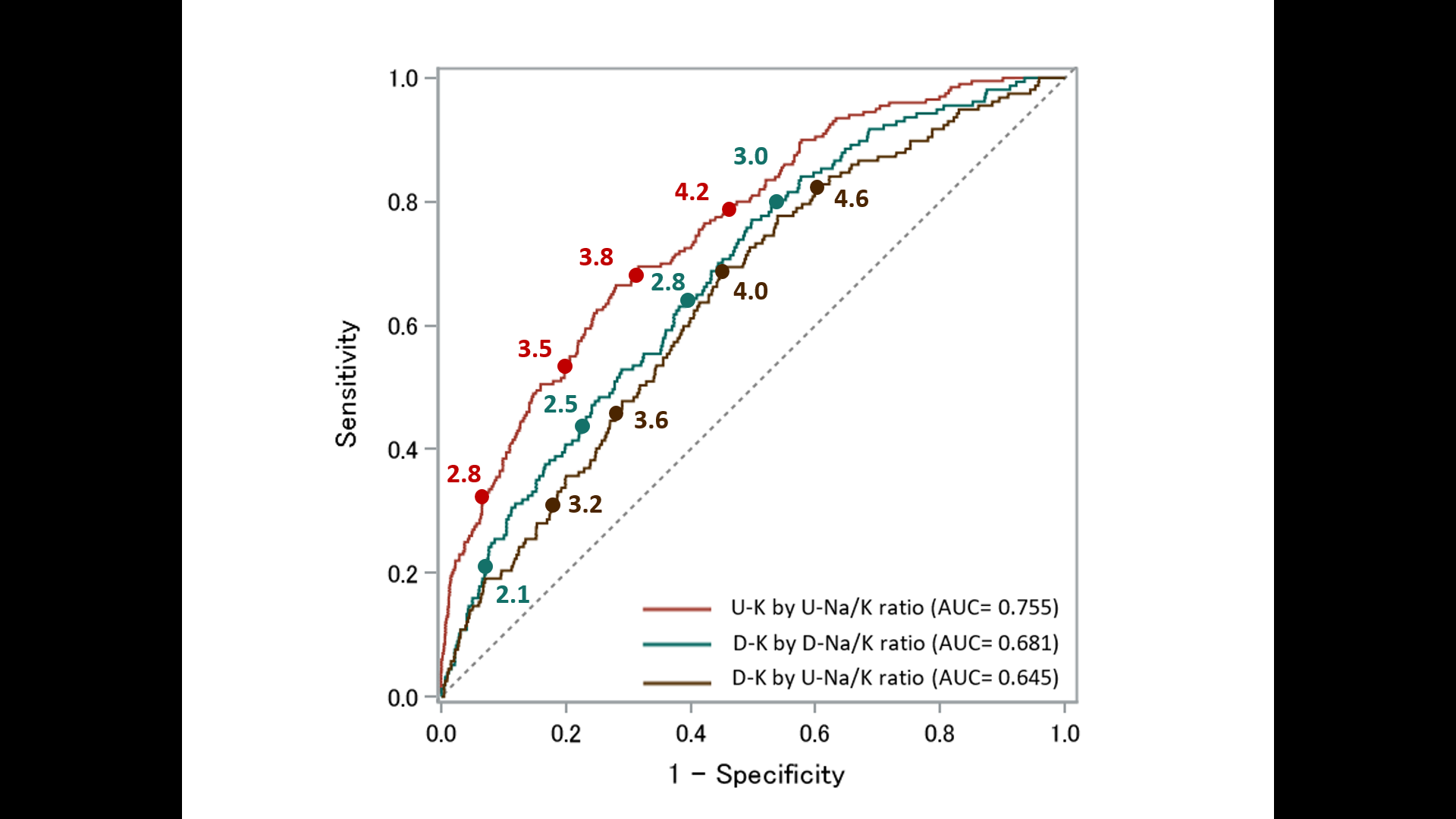


**Supplementary Fig. 3**


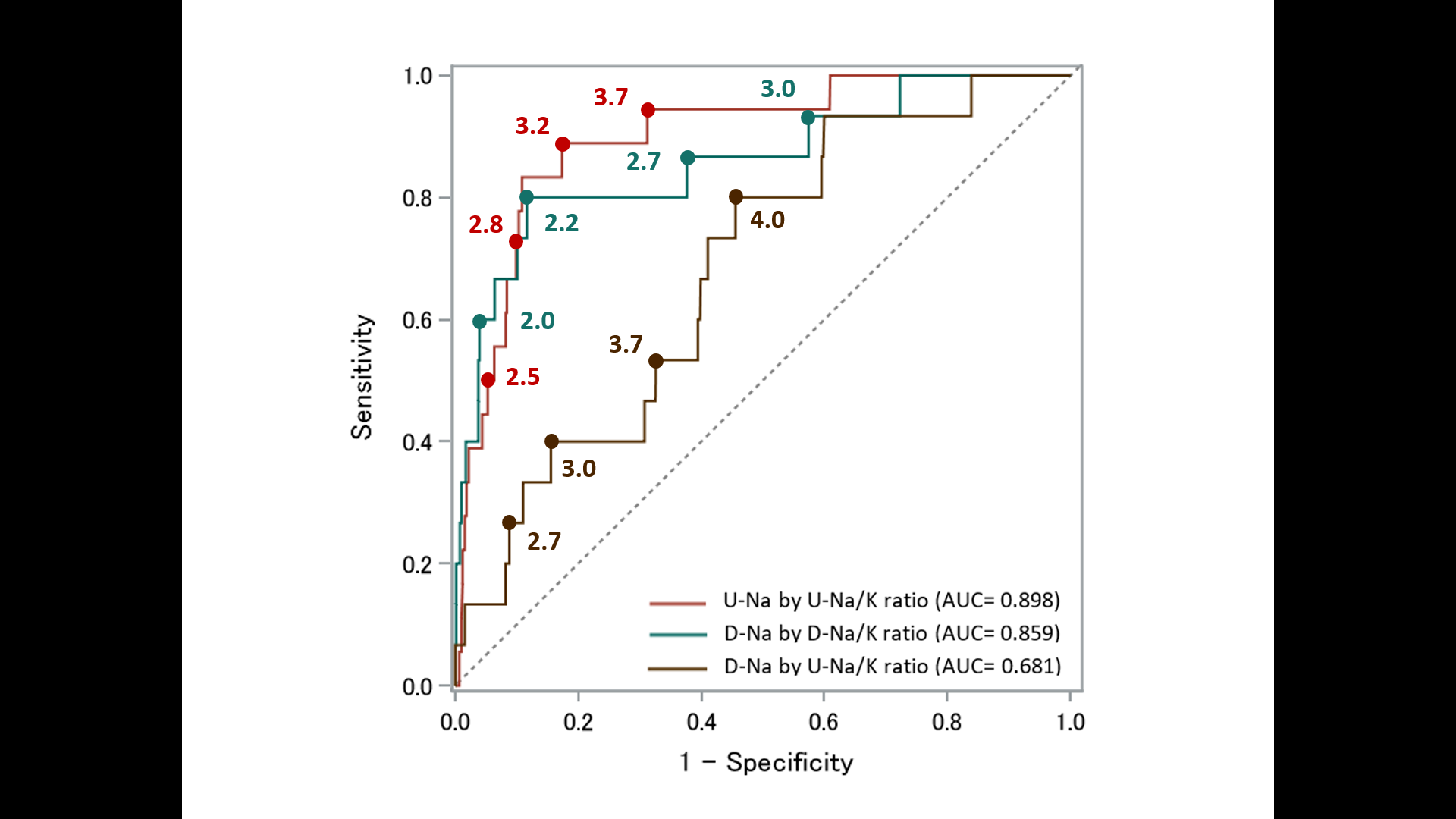

Supplement: Supplementary file 2 — Supplementary Figures [file 41440_2022_1007_MOESM2_ESM.docx]
